# After low and high dose-rate interstitial brachytherapy followed by IMRT radiotherapy for intermediate and high risk prostate cancer

**DOI:** 10.1186/s12885-016-2329-7

**Published:** 2016-05-03

**Authors:** Satoshi Nakamura, Naoya Murakami, Koji Inaba, Akihisa Wakita, Kazuma Kobayashi, Kana Takahashi, Hiroyuki Okamoto, Rei Umezawa, Madoka Morota, Minako Sumi, Hiroshi Igaki, Yoshinori Ito, Jun Itami

**Affiliations:** Department of Radiation Oncology, National Cancer Center Hospital, Chuo-ku, Tsukiji 5-1-1, Tokyo, 104-0045 Japan; Department of Radiation Oncology, Showa University Koto Toyosu Hospital, 5-1-38 Toyosu, Koto-ku, Tokyo, 135-8577 Japan; Department of Radiation Oncology, Cancer Institute Hospital of Japanese Foundation for Cancer Research, 3-8-31 Ariake, Koto-ku, Tokyo, 135-0063 Japan

**Keywords:** Clinically localized prostate cancer, Low-dose-rate brachytherapy, High-dose-rate brachytherapy, International Prostate Symptom Score (IPSS), Intensity-modulated radiation therapy (IMRT)

## Abstract

**Background:**

The study aimed to compare urinary symptoms in patients with clinically localized prostate cancer after a combination of either low-dose-rate or high-dose-rate interstitial brachytherapy along with intensity-modulated radiation therapy (LDR-ISBT + IMRT or HDR-ISBT + IMRT).

**Methods:**

From June 2009 to April 2014, 16 and 22 patients were treated with LDR-ISBT + IMRT and HDR-ISBT + IMRT, respectively. No patient from these groups was excluded from this study. The prescribed dose of LDR-ISBT, HDR-ISBT, and IMRT was 115 Gy, 20 Gy in 2 fractions, and 46 Gy in 23 fractions, respectively. Obstructive and irritative urinary symptoms were assessed by the International Prostate Symptom Score (IPSS) examined before and after treatments. After ISBT, IPSS was evaluated in the 1st and 4th weeks, then every 2–3 months for the 1st year, and every 6 months thereafter.

**Results:**

The median follow-up of the patients treated with LDR-ISBT + IMRT and HDR-ISBT + IMRT was 1070.5 days and 1048.5 days, respectively (*p* = 0.321). The IPSS-increment in the LDR-ISBT + IMRT group was greater than that in the HDR-ISBT + IMRT between 91 and 180 days after ISBT (*p* = 0.015). In the LDR-ISBT + IMRT group, the IPSS took longer time to return to the initial level than in the HDR-ISBT + IMRT group (in LDR-ISBT + IMRT group, the recovery time was 90 days later). The dose to urethra showed a statistically significant association with the IPSS-increment in the irritative urinary symptoms (*p* = 0.011). Clinical outcomes were comparable between both the groups.

**Conclusions:**

Both therapeutic modalities are safe and well suited for patients with clinically localized prostate cancer; however, it took patients longer to recover from LDR-ISBT + IMRT than from HDR-ISBT + IMRT. It is possible that fast dose delivery induced early symptoms and early recovery, while gradual dose delivery induced late symptoms and late recovery. Urethral dose reductions were associated with small increments in IPSS.

## Background

Brachytherapy is an established method in terms of both efficacy and safety for patients with localized prostate cancer [1, 2]. Interstitial brachytherapy (ISBT) for localized prostate cancer can be administered as low-dose-rate ISBT (LDR-ISBT) or high-dose-rate ISBT (HDR-ISBT). It is well known that acute urinary symptoms develop shortly after brachytherapy; this is reflected as an increase in the International Prostate Symptom Score (IPSS) [3–6]. Several reports have described the favorable efficacy of the combination of ISBT with external beam radiation therapy (EBRT) for prostate cancer [1, 2, 5, 7–10]. However, no studies have directly compared the differences in the acute urinary symptoms between these two ISBT techniques.

In our institution, a combination of intensity-modulated radiation therapy (IMRT) with either LDR-ISBT or HDR-ISBT has been applied for patients with localized prostate cancer. The current study aimed to compare the increments in IPSS after combination EBRT along with either LDR-ISBT or HDR-ISBT.

## Methods

### Patient selection

Since June 2009, ISBT for patients with clinically localized prostate cancer has been implemented in our institution. The T-stage was determined according to the International Union against Cancer (UICC) [11]. The patients were classified according to the risk classification of the National Comprehensive Cancer Network (NCCN) guidelines [12]. Patients with intermediate-risk prostate cancer with Gleason score of 4 + 3 and patients with high-risk prostate cancer were treated by a combination of IMRT of 46 Gy in 23 fractions and either LDR-ISBT of 115 Gy or HDR-ISBT of 20 Gy in 2 fractions. In our institution, HDR-ISBT + IMRT was recommended to patients with high-risk prostate cancer by treating physician because favorable clinical results have been reported after HDR-ISBT + IMRT for these patients [13, 14]. In contrast, for intermediate-risk patients, LDR-ISBT + IMRT was recommended. Based on these suggestions, the treatment method in each case was determined after discussions between the physicians and patient.

In HDR-ISBT + IMRT, because the dose delivery of HDR-ISBT requires 1–2 days, HDR-ISBT can be administered anytime during IMRT. In contrast, the dose delivery of LDR-ISBT requires several months; therefore, if LDR-ISBT is performed before IMRT, IMRT is initiated 1–2 months after LDR-ISBT is completed. If IMRT is performed earlier than LDR-ISBT, LDR-ISBT can be performed immediately after the completion of the IMRT. The numbers of patients for whom HDR-ISBT was performed before IMRT, during IMRT, and after IMRT and for whom LDR-ISBT was performed before IMRT and after IMRT were counted. The patients treated by LDR-ISBT alone were excluded from this study.

### Technique of interstitial brachytherapy

The precise technique of LDR-ISBT has been described elsewhere [4]. In brief, LDR-ISBT was performed with ^125^I seeds (Onco-Seed; Mihon Medi-Physics, Kobe, Japan) of 0.394 mCi (14.6 MBq), 0.385 mCi (14.2 MBq), or 0.416 mCi (15.4 MBq) under general anesthesia. No margins were added around the prostate (clinical target volume = planning target volume). At 1 month after LDR-ISBT, post-plan dosimetry was performed in all patients [15–17]. Computed tomography (CT) images of 2-mm thickness were taken at 2-mm intervals with a Foley catheter in place. T2-weighted magnetic resonance images (MRI) were also obtained on the same day with a Foley catheter and fused with the CT images to ensure precise contouring of the prostate.

In HDR-ISBT, plastic catheters were inserted under general and epidural anesthesias with the guidance of TRUS using the perineal template. After catheter placement, CT of the implanted region was performed by a large bore CT simulator (Aquilion™, Toshiba, Tokyo, Japan) with the patient lying in the lithotomy position. As in LDR-ISBT, 2-mm thick CT images were taken with 2-mm intervals. The prostate, urethra, rectum, and bladder were contoured and stored in Oncentra® (ver. 4.1, Nucletron, Veenendaal, The Netherlands). As in the case with LDR-ISBT, no margins were added to the prostate. HDR-ISBT was carried out by ^192^Ir source remote afterloading system (RALS, MicroSelectron HDR™, Nucletron, Veennendaal, The Netherlands), with ^192^Ir activity of approximately 10 Ci [18, 19]. The prescription dose of HDR-ISBT was 20 Gy in 2 fractions with a 6-h interval with patients lying on the bed during the treatment.

The number of dwell positions in HDR-ISBT and ^125^I seeds in LDR-ISBT was counted because the dose distribution was related to these numbers.

### Technique of intensity-modulated radiation therapy

IMRT was performed with either the Volumetric Modulated Arc Therapy (VMAT) technique or Sliding-window technique with a linear accelerator (Clinac iX; Varian Medical Systems) using 15-MV photon beams. Treatment planning for IMRT was based on CT images of 2-mm slice thickness with 2-mm intervals obtained with a large bore CT simulator and calculated by Eclipse (ver. 8–11, Varian Medical Systems). MRI and CT images were fused to decide a target definition. However, images from positron emission tomography (PET) were not used for the target definition. Three different types of plans were made as follows: (a) the clinical target volume (CTV) was defined as the prostate, whole seminal vesicle and regional pelvic lymph nodes; (b) the CTV was defined as the prostate and whole seminal vesicle; and (c) the CTV was defined as the prostate plus the proximal one-third of the seminal vesicle. Indications for plan (a) were as follows: patients having two of the following high risk factors: T3a, level of prostate-specific antigen (PSA) > 20 ng/dL, Gleason score ≥ 8, or patients with T3b. The indication for plan (b) was patients with T3b. All the remaining patients were treated by plan (c). The planning target volume (PTV) for the prostate in the (a) plan was defined as the CTV plus 10 mm in the lateral, anterior, and cranio-caudal directions as well as 7 mm in the posterior direction. The PTV in plans (b) and (c) plans was defined as the CTV plus 5 mm in the left-right, anterior, and cranio-caudal directions as well as 4 mm in the posterior direction. A greater PTV margin was used in plan (a) because the patients were aligned using the bony structures to ensure the proper positioning of the pelvic lymphatic node area. In plan (b) or (c), on the other hand, a smaller PTV margin was applied because the daily movement of the prostate was tracked by abdominal ultrasonography or an electric portal imaging device for patients with gold markers in the prostate. The numbers of patients treated as per the (a), (b), and (c) plans were counted for each treatment.

The same dose constraints applied for patients treated with IMRT alone (78 Gy in 39 fractions) were applied for the patients included in our study (IMRT: 46 Gy in 23 fractions) with some modifications. The IMRT plan of 46 Gy in 23 fractions was converted into an IMRT plan of 78 Gy in 39 fractions to evaluate the dose constraints, and the following dose constraints were applied: no more than 60 % and 35 % of the volume of the bladder wall were to receive a dose greater than 40 Gy and 70 Gy, respectively, and no more than 60 %, 35 %, 25 %, and 1 % of the volume of the rectal wall were to receive a dose greater than 40 Gy, 60 Gy, 70 Gy, and 80 Gy, respectively. In all the patients, the dose for each organ at risk (OAR) passed the dose constraints while maintaining the coverage of PTV.

### Dose evaluation

Dose distribution of the LDR-ISBT, HDR-ISBT, and IMRT was calculated with VariSeed™, Oncentra™, and Eclipse™, respectively. In order to compare the dose distributions of LDR-ISBT + IMRT with that of HDR-ISBT + IMRT, the equivalent doses in 2 Gy/fraction (EQD_2_) for IMRT, LDR-ISBT, and HDR-ISBT were calculated by rewriting the DICOM-RT by using Python (x,y)™ (ver. 2.7.6). The EQD_2_ calculation of the LDR-ISBT was given by equation (1) [20], and that of HDR-ISBT as well as IMRT was given by equation (2) [20].

1 $$ \begin{array}{l}EQ{D}_2=D\frac{\left(\frac{R_0}{\mu +\lambda }+\alpha /\beta \right)}{\left(2+\alpha /\beta \right)}\\ {}\end{array} $$

2 $$ EQ{D}_2=nd\frac{\left(d+\alpha /\beta \right)}{\left(2+\alpha /\beta \right)} $$

where *D* is the accumulated dose, *R*_*0*_ is the initial dose rate, λ is the radioactive decay constant, μ is the rate of repair of sub-lethal damage, n is the number of fractions, and d is the dose per fraction. Because acute urethral complications were investigated in this study, the α/β ratio used in this study was 10 Gy, μ was 0.462 h^−1^, and λ was 4.86 × 10^−4^ h^−1^ [21].

After the rewriting of the DICOM-RT, the DICOM-RT was transferred from each treatment planning system (TPS) to the MIM Maestro™ software (ver. 6, MIM software, OH, USA). Then, the LDR-ISBT dose and the IMRT dose or the HDR-ISBT dose and IMRT dose were summed using MIM Maestro™.

The urethra was contoured as the outer rim of the Foley catheter from the bladder neck to the most caudal prostate that could be found. In addition to the urethra, the basal urethra was defined as the most proximal one-third of the prostatic urethra in proximity to the bladder trigone and contoured as an OAR for this study. Although the relationship between the dose to the bladder trigone and increments in the IPSS was investigated by the MSKCC group [22], it was difficult to evaluate the dose to the bladder trigone in this study, because the patient’s position in the CT-images at HDR-ISBT did not correspond to those of IMRT, and the CT coverage during ISBT was not adequate in the cranial direction in order to identify the ureteral orifices. Therefore, in this study, the base of the urethra was evaluated as a surrogate structure for the bladder trigone.

The registration of anatomic structures contoured on different CT series of ISBT and IMRT was performed on the basis of the contouring of the urethra and prostate by Eclipse™. The evaluations of the cumulative dose to the whole urethra and the base of the urethra were performed by the CT image for the IMRT planning. The dose-volume histogram (DVH) was examined in 0.1 Gy steps. In IMRT planning, the dose to the urethra was analyzed to evaluate the variances in the dose to the urethra.

### Urinary symptoms

The increment in IPSS was defined as the difference between the IPSS before (initial IPSS) and after the ISBT. Recovery time was defined as the time from the completion of the radiation therapy to the time point when the difference between the initial and after-the-treatment IPSS values lost its significance after the maximum increment in the IPSS. After ISBT, in general, IPSS was evaluated in the 1st and 4th weeks, then every 2–3 months for the 1st year, and every 6 months thereafter. The IPSS consists of 7 questions classified into either obstructive (Items 1, 3, 5, and 6) or irritative (Items 2, 4, and 7) symptoms [23]. Therefore, not only IPSS as a total score (t-IPSS) but also the scores for obstructive symptoms (o-IPSS) and irritative symptoms (i-IPSS) were also investigated separately.

Because Ghadjar et al. showed that an increment in IPSS greater than 10 points from the initial IPSS was related to the dose to the bladder trigone [22], the analysis in the present study included the following endpoints: increment from initial t-IPSS + 10 endpoint, the initial o-IPSS + 5 endpoint, and the initial i-IPSS + 5 endpoint.

### Statistical analysis

The relationship between clinical and treatment variables and the increment in IPSS was analyzed by univariate analysis. The variance was analyzed by Shapiro-Wilk test to detect the variance of distribution. As a result, if the variance of distribution of each IPSS was normal, we used Student’s *t*-test. On the other hand, if the variance of distribution was not normal, we used the Mann–Whitney-*U* test. The *t*-test was used to compare continuous variables, and Pearson *χ*^2^ test was used to compare categorical variables. Time to overall survival (OS), biochemical progression free survival (BPFS), and progression free survival (PFS) were analyzed with Kaplan-Meier method, and the log-rank test was performed. The biochemical control rate was defined with using Phoenix criteria [24]. A *p*-value < 0.05 was considered as statistically significant. All continuous clinical variables and DVH parameters were dichotomized at the median value and analyzed. Multiple logistic regression analysis was performed using the variables that showed significant difference in the univariate analysis.

This retrospective study was approved by the institutional review board of the National Cancer Center (2014–223). The informed consent was not taken from each patient because this retrospective study was approved by institutional ethical committee and it was decided that the ethic committee waived taking informed consent from each patient.

## Results

### Patients

From June 2009 through April 2014, 16 and 22 patients were treated with the combination of LDR-ISBT plus IMRT and HDR-ISBT plus IMRT (LDR-ISBT + IMRT or HDR-ISBT + IMRT), respectively. No patient was excluded from this study. Clinical characteristics of the patients are summarized in Table 1. Three patients in the LDR-ISBT + IMRT group received neoadjuvant androgen deprivation therapy (ADT), while 11 in the HDR-ISBT + IMRT group received ADT. After ISBT, ADT was stopped unless patients experienced biochemical or clinical recurrence. In the HDR-ISBT + IMRT group, the number of patients for whom HDR-ISBT was performed before IMRT, during IMRT, and after IMRT was 11, 6, and 5, respectively. The pretreatment level and increment in i-IPSS showed significant differences among three treatment sequencings (*p* < 0.05). In the LDR-ISBT + IMRT group, the number of patients for whom LDR-ISBT was performed before IMRT and after IMRT was 14 and 2, respectively. There was no significant difference in the pretreatment level and increments in the t-IPSS, o-IPSS, and i-IPSS (*p* > 0.05). The number of dwell positions in HDR-ISBT and the ^125^I seeds in LDR-ISBT was 265.7 ± 103.2 and 66.1 ± 16.1 (*p* < 0.001), respectively. Among the patients who received HDR-ISBT + IMRT, the number of patients with CTV (a), (b), and (c) was 11, 8, and 3, respectively, while among the patients who received LDR-ISBT + IMRT, it was 1, 12, and 3, respectively. In the HDR-ISBT + IMRT group, the pretreatment level of i-IPSS showed a significant difference among the 3 CTV definitions (*p* = 0.04). However, an increment in t-IPSS, o-IPSS, and i-IPSS showed no significant differences among the 3 CTV definitions (*p* > 0.05). Among the patients who received LDR-ISBT + IMRT, there were no significant differences in the pretreatment level or the increments in t-IPSS, o-IPSS, and i-IPSS among the 2 CTV definitions ((b) and (c); *p* > 0.05).

**Table 1:** Patient characteristics

	HDR-ISBT+IMRT	LDR-ISBT+IMRT	*p* value
	*n*(%)	*n*(%)	
Age[years], median(range)	67.8 (54.5-81.4)	65.8 (52.6-78.8)	0.498
Stage			0.867
■T1b	1 (4.5)	1 (6.3)	
■T1c	11 (50)	9 (56.2)	
■T2a	2 (9.1)	1 (6.3)	
■T2b	2 (9.1)	3 (18.8)	
■T2c	2 (9.1)	1 (6.3)	
■T3a	2 (9.1)	1 (6.3)	
■T3b	2 (9.1)	0 (0)	
Initial PSA			0.061
■<10	7 (31.8)	8 (50)	
■ 10-20	8 (36.4)	7 (43.7)	
■>20	7 (31.8)	1 (6.3)	
Gleason Score			0.029*
■<7	0 (0)	0 (0)	
■7	17 (77.3)	16 (100)	
■>7	5 (22.7)	0 (0)	
Risk grouping (NCCN classification)			0.031*
■ Low	0 (0)	0 (0)	
■ Intermediate	12 (54.5)	14 (87.5)	
■ High	10 (45.5)	2 (12.5)	
Baseline t-IPSS, median(range)	8.3 (1.0-23.0)	6.75 (1.5-23.0)	0.982
ADT	10 (45.5)	3 (18.8)	0.087
Folow-up [days], median(range)	1048.5 (409–2199)	1070.5 (617–2199)	0.321
Prostate volume [cc], median(range)	40.8(17.5-94.5)	33.1(13.6-73.8)	0.12

*Abbreviations*: *HDR-ISBT + IMRT* combination of HDR-ISBT and intensity-modulated radiation therapy, *LDR-ISBT + IMRT* combination of LDR-ISBT and intensity-modulated radiation therapy, *ADT* androgen deprivation therapy, *NCCN* National Comprehensive Cancer Network

### Urinary symptoms

**Fig. 1:**
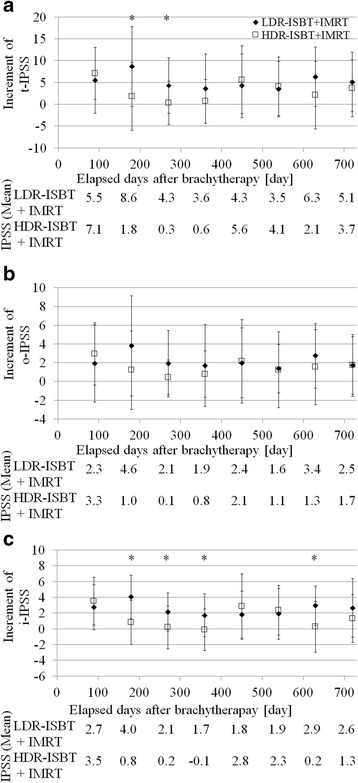
Time change of the International Prostate Symptom Score (IPSS). **a** A total score after interstitial brachytherapy (t-IPSS), and **b** the obstructive symptom (o-IPSS), and **c** the irritative symptom (i-IPSS). The * indicates a period that has a statistically significant difference

The mean initial t-IPSS of the LDR-ISBT + IMRT and HDR-ISBT + IMRT groups was 9.48 and 9.53, respectively (*p* = 0.983). The mean initial o-IPSS of the LDR-ISBT + IMRT and HDR-ISBT + IMRT groups was 5.31 and 4.64 (*p* = 0.677), while the mean initial i-IPSS was 4.17 and 4.91, respectively (*p* = 0.429). The t-IPSS, o-IPSS, and i-IPSS in the HDR-ISBT + IMRT group reached its maximum 0–90 days after HDR-ISBT, while that in the LDR-ISBT + IMRT group reached its maximum 91–180 days after LDR-ISBT. A significant difference between the LDR-ISBT + IMRT and HDR-ISBT + IMRT was found in the increments in the t-IPSS during 91–180 and 181–270 days (Fig. 1a; *p* = 0.015 and 0.037, respectively), and in the increment in the i-IPSS (*p* = 0.013,- 0.015) during 91–180, 181–270, 271–360, and 541–630 days (Fig. 1c; *p* = 0.001, 0.027, 0.013, and 0.015, respectively). However, no significant differences were noted in the increments in the o-IPSS (Fig. 1b). In t-IPSS, the recovery time in the LDR-ISBT + IMRT and HDR-ISBT + IMRT groups were 181–270 days and 91–180 days, respectively. In o-IPSS, the recovery time in the LDR-ISBT + IMRT and HDR-ISBT + IMRT groups were 181–270 days and 91–180 days, respectively (Fig. 1b). In i-IPSS, the recovery time of the LDR-ISBT + IMRT and HDR-ISBT + IMRT groups were 361–450 days and 91–180 days, respectively (Fig. 1c).

**Fig. 2:**
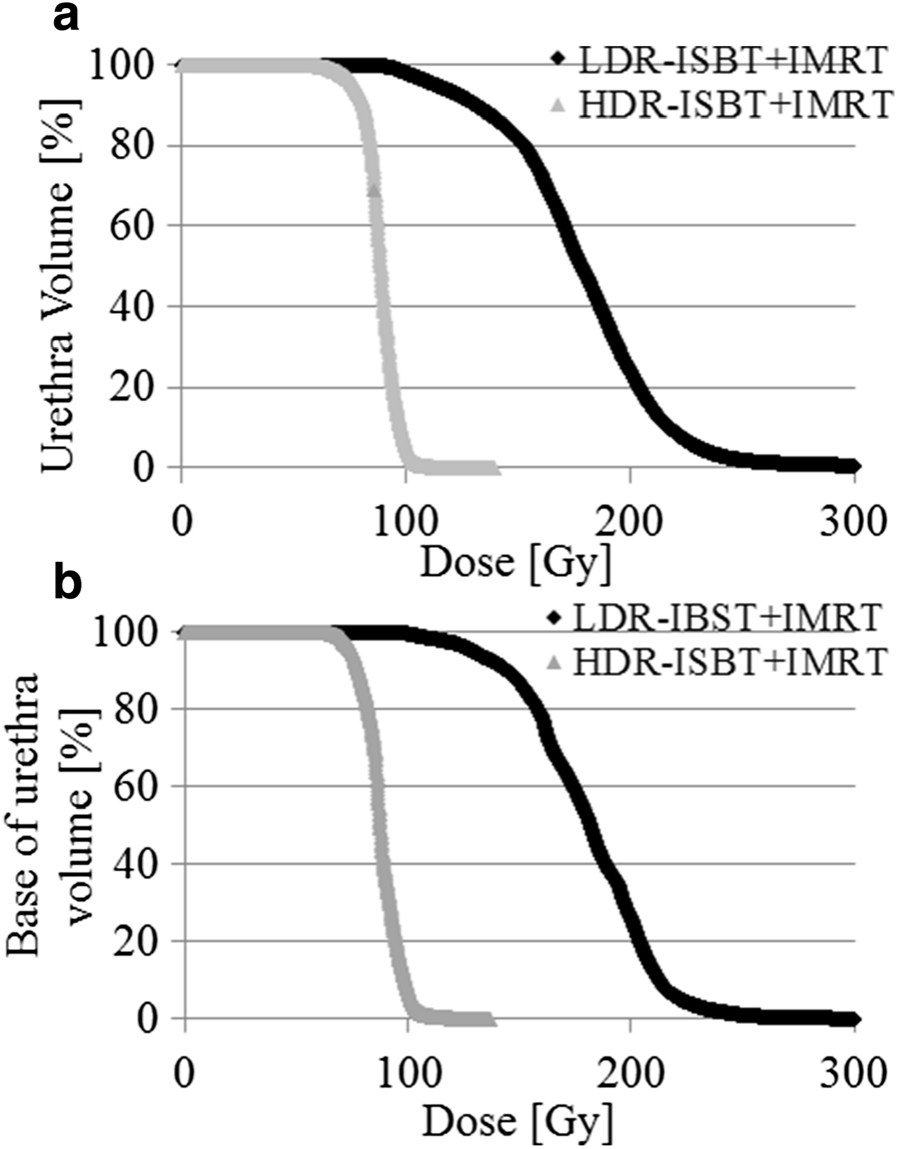
Dose volume histogram. **a** Dose volume histogram of the urethra, and **b** Dose volume histogram of the base of urethra

With respect to the DVH, a significant difference was found in both the volume of the urethra and base of the urethra receiving more than 69.4 Gy in EQD2 and 74.3 Gy in EQD2 between the patients receiving LDR-ISBT + IMRT and HDR-ISBT + IMRT (Fig. 2a and b, *p* < 0.05). The dose to the prostate delivered by the IMRT component in LDR-ISBT + IMRT and HDR-ISBT + IMRT was 46.5 ± 1.0 Gy and 46.7 ± 1.1 Gy ((mean dose) ± σ; *p* = 0.311), respectively.

The results for the univariate analysis for the increments from initial t-IPSS + 10 endpoint, the initial o-IPSS + 5 endpoint, and the initial i-IPSS + 5 endpoint are summarized in Table 2. The D_50%_ of the urethra was associated with the initial t-IPSS + 10 and the initial i-IPSS + 5 endpoints (*p* = 0.024, and 0.031, respectively). The brachytherapy technique, the D_50%_ of the base of the urethra, the V_90_ of the urethra and base of the urethra, and the V_100_ of the urethra were also associated with the i-IPSS +5 endpoint (*p* < 0.05).

**Table 2:** The univariate analysis for IPSS increment. The univariate analysis of wheather t-IPSS increased by ten or over ten points during 91–180 days after ISBT. Similary, o-IPSS and i-IPSS increment of five or over five points during the same time period was analyzed

*p* value				
Factor		t-IPSS+10	o-IPSS+5	i-IPSS+5
Age	<66.17 vs >66.17	0.838	0.959	0.253
Prostate volume	<36.7 ml vs >36.7 ml	0.681	0.457	0.750
Risk group	intermediate vs high	0.820	0.540	0.318
Initial PSA	<11.84 vs >11.84	1.000	0.609	0.124
Baseline t-IPSS	<7.67 vs >7.67	0.217	0.400	0.253
ADT	yes vs no	1.000	0.505	0.472
Brachytherapy technique	LDR-ISBT vs HDR-ISBT	0.066	0.193	0.010^*^
D_50_ % of urethra	<95.65 Gy vs >95.65 Gy	0.024^*^	0.101	0.031^*^
D_50_ % of base of urethra	<95.70 Gy vs >95.70 Gy	0.152	0.397	0.031^*^
V90 of urethra	<85.32 % vs >85.32 %	0.152	0.397	0.031^*^
V_90_ of base of urethra	<75.01 % vs >75.01 %	0.152	0.397	0.031^*^
V100 of urethra	<17.05 % vs >17.05 %	0.217	0.535	0.005^*^

*Abbreviations*: *ADT* androgen deprivation therapy, *IPSS* international Prostate Symptom Score, *t-IPSS* total score of IPSS, *o-IPSS + 5* total score of IPSS about obstructive symptom, *i-IPSS + 5* total score of IPSS about irritative symptom, *Dx%* minimum dose delivered to x% of the organ volume, *Vx* proportion of volume receiving x Gy. The Gy indicates the dose which was converted into the EQD2

The * indicates a variable that has a significant difference

The results of the multiple logistic regression analysis are shown in Table 3. The D_50%_ of the urethra was a predictor for the initial i-IPSS + 5 (*p* = 0.011).

**Table 3:** The multiple logistic regression analysis for increment of IPSS. The multiple logistic regression analysis of whether t-IPSS incresased by ten or over ten points during 91–180 days after ISBT. Similary, i-IPSS increment of five or over five points during the same time period was analyzed

IPSS	Factor	Odds ratio (95 % of confidence level)	*p* value
t-IPSS + 10	D50 % of urethra	1.021 (1.001-1.041)	0.035*
	D50 % of urethra	1.039 (1.009-1.069)	0.011^*^
	D50 % of base of urethra	-	0.868
i-IPSS + 5	V90 of urethra	-	0.657
	V90 of base of urethra	-	0.411
	V100 of urethra	-	0.427
	Brachytherapy technique	-	0.100

t-IPSS + 10 (D_50_ % of urethra): Model x test: p<0.001, Determine predictive value: 80.6 %; i-IPSS + 5 (D_50_ % of urethra): Model x test: p<0.001, Determine predictive value: 90.3 %

*Abbreviations*: *IPSS* international Prostate Symptom Score, *t-IPSS* total score of IPSS, *i-IPSS* total score of IPSS about irritative symptom, *Dx%* minimum dose delivered to x% of the organ volume, *Vx* proportion of volume receving x Gy. The Gy indicates the dose which was converted into the EQD2

The * indicates a variable that has a significant difference

None of the patients in this study experienced urinary tract infection.

### Rectal symptoms

In the HDR-ISBT + IMRT and LDR-ISBT + IMRT groups, 0 and 2 patients, respectively, developed grade 2 rectal bleeding according to Common Toxicity Criteria (*p* = 0.088).

### Clinical outcome

**Fig. 3:**
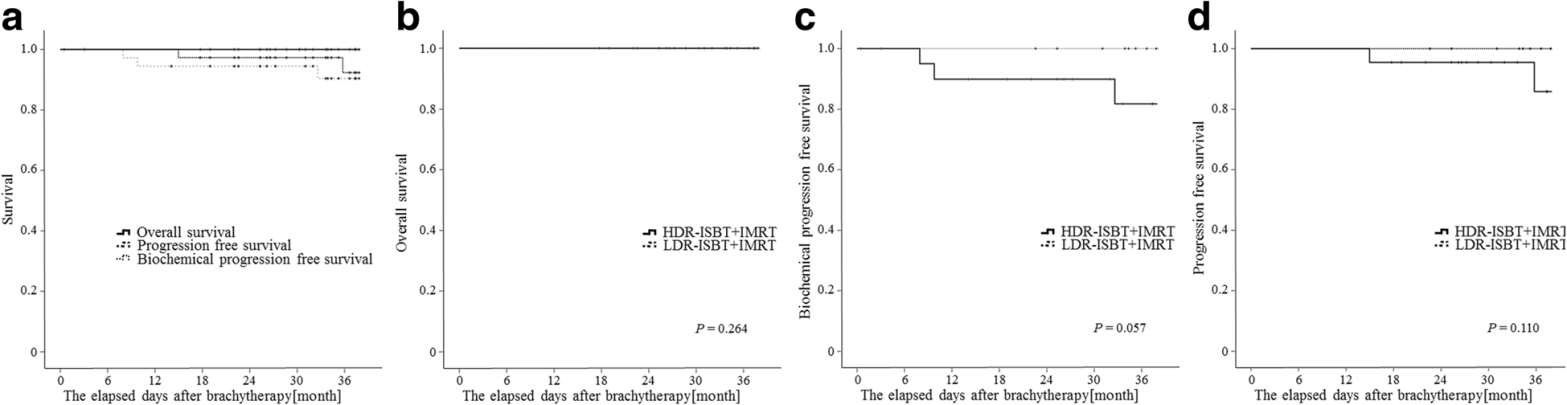
Kaplan-Meier curves of **a** Survival, **b** Overall survival, **c** Biochemical progression free survival, and **d** Progression free survival

The 3-year OS rate, BPFS rate, and PFS rate for all the patients included in the current study were 97.4 %, 89.5 %, 92.1 %, respectively (Fig. 3(a)). In the HDR-ISBT + IMRT group, only 1 patient died. In the LDR-ISBT + IMRT group, no patient died during the study period. The number of patients who suffered biochemical failure (PSA failure) in the HDR-ISBT + IMRT and LDR-ISBT + IMRT groups was 4 and 0, respectively. The number of patients with clinical recurrence in the HDR-ISBT + IMRT and LDR-ISBT + IMRT groups was 3 and 0, respectively. In the HDR-ISBT + IMRT and LDR-ISBT + IMRT groups, the 3-year OS, BPFS, and PFS were 100 %, 86.4 %, and 90.9 % and 100 %, 100 %, and 100 %, respectively (Fig. 3(b), (c), and (d); *p* = 0.264, 0.057, and 0.110, respectively). Figure 3 shows the Kaplan-Meier curves for OS, BPFS, and PFS.

## Discussion

In this study, the direct comparison of the IPSS between the LDR-ISBT + IMRT and HDR-ISBT + IMRT groups showed that the increments in IPSS among the patients receiving LDR-ISBT + IMRT occurred later than that in the HDR-ISBT + IMRT group. In the LDR-ISBT + IMRT group, the maximal increase in the t-IPSS occurred around 90–180 days after LDR-ISBT and the IPSS returned to the pretreatment level between 181 and 270 days. The timing of the maximal increase in IPSS in the LDR-ISBT group was in accordance with previous findings, although that study focused on patients treated only with LDR-ISBT [4]. Murakami et al. reported that the timing of the maximum increase in t-IPSS in LDR-ISBT was 3 months after ISBT [4]; therefore, it was likely that the maximum increase in the LDR-ISBT + IMRT group was observed later than that in LDR-ISBT alone because IMRT was additionally performed. The maximum increments in t-IPSS in the study by Murakami et al. and in the present study were 10.7 ± 6.9 and 8.6 ± 9.2 ((mean) ± (1 SD)) [4], respectively. In contrast, in the HDR-ISBT + IMRT group, the maximal increase in the IPSS occurred around 0–90 days after HDR-ISBT and the IPSS returned to the pretreatment level between 91 and 180 days. Mahmoudieh et al. reported that the timing of the maximum increase in t-IPSS in HDR-ISBT was 6 weeks, and the t-IPSS returned to the pretreatment level after 6 months [5]. These results corresponded with those of the current study. The maximum increment in t-IPSS in the study by Mahmoudieh et al. was approximately 4 (mean), while that in our study was 7.1 ± 6.0 ((mean) ± (1 SD)). In LDR-ISBT, 98.6 % of the prescription dose is delivered over a period as long as 1 year and 65.5–87.8 % of the dose delivery is completed by 91–180 days after the initiation of LDR-ISBT. In contrast, in HDR-ISBT, the prescription dose is delivered within only 1–2 days. This huge difference in the total duration of dose delivery between LDR-ISBT and HDR-ISBT may have an enormous influence on the differences observed in the timing of increment and recovery of IPSS in patients treated with LDR-ISBT and HDR-ISBT.

The structure of the urethra on the CT series during IMRT was not contoured precisely because the Foley catheter was not inserted when CT images for IMRT were taken. However, this issue was not important because in LDR-ISBT + IMRT and HDR-ISBT + IMRT both, the dose to the prostate delivered by the IMRT component was approximately the same (*p* = 0.311) because the urethra and base of the urethra was defined as the prostatic urethra. As a result, the contouring uncertainty of the urethra could be ignored.

The IPSS consists of two distinct urinary symptom categories, i.e., obstructive and irritative symptoms, and the present study assessed both categories in detail. In the current study, multivariate analysis revealed that the increments in the IPSS related to irritative symptoms, for which the responsible organ was supposed to be the bladder, were related to the D_50 %_ of the urethra. In contrast, univariate analysis demonstrated that the D_50 %_ of the base of the urethra, the V_90_ of the urethra and base of the urethra, the V_100_ of the urethra, and the brachytherapy technique were related with i-IPSS + 5. Member of the patient groups in the D_50 %_ of the base of the urethra and the V_90_ of the base of the urethra were the same. As a result, the same *p*-value was calculated. On the other hand, member in the D_50 %_ of the urethra and the V_90_ of the urethra was different from those groups, and member in the D_50 %_ of the urethra was also different from that in V_90_ of the urethra. The D_50 %_ of the urethra was a unique patients group. Therefore, although *p*-value was the same in i-IPSS + 5, the D_50 %_ of the urethra in i-IPSS + 5 alone showed a significant difference in multivariate analysis. Although the base of the urethra was not be found to be a predictive factor in multivariate analysis, it showed a significant difference in the univariate analysis. Thus, since the base of the urethra was intended to be used as a surrogate structure for the bladder trigone in the current study, this finding might be in line with the results of Ghadjar et al. that the bladder trigone was responsible for the increment in IPSS after IMRT [22]. Further, the D_50%_ of the urethra was related to the increment in t-IPSS + 10 points, although Ghadjar et al. reported that a maximal dose to the bladder trigone of over 90.9 Gy was related to the increment of t-IPSS + 10 points [22]. Taken together, these results suggests that the severity of acute urinary morbidities, as represented by increments in IPSS was lowered by reducing the dose to the urethra (Figs. 1 and 2, and Tables 2 and 3). This finding is in line with anatomical distribution of the autonomic nerve of the bladder. Recently, Sprandling et al. reported using cadavers with 3-dimensional image reconstruction that bladder autonomic nerves are located in the posterior region of the prostatic urethra in the male [25]. Technically, in HDR-ISBT, the dose to the urethra can be easily decreased because the dwell positions in HDR-ISBT are more than the number of ^125^I seeds used in LDR-ISBT.

Favorable clinical results have been reported for both LDR-ISBT + EBRT and HDR-ISBT + EBRT for prostate cancer [7, 9]. Similarly, our results indicated no significant difference in the clinical outcomes, i.e., OS, BPFS, and PFS, between the LDR-ISBT + IMRT and HDR-ISBT + IMRT groups (Fig. 3). The advantage of LDR-ISBT is the short procedure time, while its disadvantages are the long recovery time and the trend of more severe acute urinary symptoms as compared to HDR-ISBT, as shown in the present study. The advantages of HDR-ISBT include the short recovery time and less severe urinary symptoms; moreover, HDR-ISBT easily allows dose adjustment for each organ. However, the disadvantage of HDR-ISBT is that patients are confined to the hospital bed for at least 6-h while the applicator needles are in place. Thus, both the ISBT techniques have their advantages and disadvantages that are not related to overall clinical outcomes; therefore, the treatment method should be selected in each case after detailed discussion between the attending physician and the patient.

This study had certain limitations (e.g., retrospective analysis, small sample size, different sequencing of ISBT, different distributions of risk groups, different Gleason scores, differences in the distributions of ADT, and no standardized protocol). It has been reported that various parameters (e.g., initial IPSS or neoadjuvant hormone therapy) are related to increments in IPSS. However, these relatiofnships have not been established thus far [4, 26]. In the present study, even if the TNM stage, risk categories, and Gleason scores differed between the two groups, our aim was not to compare clinical results but rather the treatment-related toxicities between the two groups; therefore, it was considered feasible to compare groups of patients with different backgrounds. Moreover, it was likely that differences in the CTV did not influence the increments in IPSS. However, it would not be appropriate to discuss the relationship between different CTV definitions and increments in IPSS because only 1 patient in the LDR-ISBT + IMRT group was treated for plan (a). In the different sequencings, although the increments in i-IPSS showed significant differences among the three sequencing in the HDR-ISBT + IMRT group, it was likely that the significant differences pertained to the significant differences in the pretreatment level of i-IPSS. In the LDR-ISBT + IMRT, there was no significant difference in pretreatment levels or increments in t-IPSS, o-IPSS, and i-IPSS (*p* > 0.05). Therefore, the difference in treatment sequencing may not have influenced the increments in IPSS; however, we did not consider it appropriate to discuss this relationship because of the limited number of patients treated with IMRT followed by LDR-ISBT.

## Conclusions

This study was the first to perform a direct comparison of IPSS between LDR-ISBT and HDR-ISBT for patients with localized prostate cancer. Increments in IPSS in the HDR-ISBT + IMRT group occurred sooner than in the LDR-ISBT + IMRT group. Further, patients treated with HDR-ISBT + IMRT showed a shorter recovery time than those treated in with LDR-ISBT + IMRT with respect to urinary symptoms. It is possible that fast dose delivery induced early symptoms and early recovery, while gradual dose delivery induced late symptoms and late recovery. Our findings also indicated that the increment in the total IPSS and the IPSS concerning the irritative symptoms was related to the D_50%_ of the urethra. Therefore, urethral dose reductions were associated with small increments in IPSS.

### Ethics approval and consent to participate

This retrospective study was approved by the institutional review board of the National Cancer Center (2014–223).

### Consent for publication

The informed consent was not taken from each patient because this retrospective study was approved by institutional ethical committee and it was decided that the ethic committee waived taking informed consent from each patient.

### Availability of data and materials

The datasets supporting the conclusions of this article are included within the article.

## Abbreviations

ADT: androgen deprivation therapy
ADT: neoadjuvant androgen deprivation therapy
BPFS: biochemical progression free survival
CT: Computed tomography
CTV: clinical target volume
DVH: Dose volume histogram
D_x_%: minimum dose delivered to x%
EBRT: external beam radiation therapy
EQD_2_: Equivalent dose in 2 Gy/fraction
HDR-ISBT: High-dose-rate interstitial brachytherapy
HDR-ISBT + IMRT: combination of high-dose-rate interstitial brachytherapy and intensity-modulated radiation therapy
i-IPSS: Partial IPSS about irritative symptom
i-IPSS + 5: Total score of baseline IPSS + 5 point about irritative symptom
IMRT: intensity-modulated radiation therapy
initial IPSS: IPSS before interstitial brachytherapy
IPSS: International Prostate Symptom Score
ISBT: interstitial brachytherapy
LDR-ISBT: low-dose-rate interstitial brachytherapy
LDR-ISBT + IMRT: combination of low-dose-rate interstitial brachytherapy and intensity-modulated radiation therapy
MRI: magnetic resonance images
NCCN: National Comprehensive Cancer Network
OAR: organ at risk
o-IPPSS + 5: total score of baseline IPSS + 5 point about obstructive symptom
o-IPSS: Partial IPSS about obstructive symptom
OS: overall survival
PFS: progression free survival
PSA: prostate-specific antigen
PTV: planning target volume
t-IPSS: total score of IPSS
t-IPSS + 10: Total score of baseline IPSS + 10 point
TPS: treatment planning system
TRUS: trans-rectal ultrasonography
UICC: International Union against Cancer
VMAT: Volumetric Modulated Arc Therapy
V_x_: proportion of volume receiving x Gy

## References

[1] Stone NN, Stock RG, Cesaretti JA, Unger P (2010). Local control following permanent prostate brachytherapy: effect of high biologically effective dose on biopsy results and oncologic outcomes. Int J Radiat Oncol Biol Phys.

[2] ÅstrÖm L, Pedersen D, Mercke C, Holmäng S, Ohanssoon KA (2005). Long-term outcome of high dose rate brachytherapy in radiotherapy of localized prostate cancer. Radiationther Oncol.

[3] Barry MJ, Fowler FJ, O’Leray MP, Bruskewitz RC, Holtgrewe HL, Mebust WK (1992). The American Urological Association symptom index for benign prostatic hyperplasia. The measurement Committee of the American Urological Association. J Urol.

[4] Murakami N, Itami J, Okuma K, Hiroshi M, Keiichi N, Tsukasa B (2008). Urethral doseand increment of international prostate symptom score (IPSS) in transperineal permanent interstitial implant (TPI) of prostate cancer. Strahlenther Onkol.

[5] Mahmoudieh A, Tremblay C, Beaulieu L, Lachance B, Harel F, Lessard E (2005). Anatomy-based inverse planning dose optimization in HDR prostate implant: a toxicity study. Radiother Oncol.

[6] Hoskin P, Rojas A, Ostler P, Hughes R, Alonzi R, Lowe G (2014). High-dose-rate brachytherapy alone given as two or one fraction to patients for locally advanced prostate cancer: Acute toxicity. Radiother Oncol.

[7] Kotecha R, Yamada Y, Pei X, Kollmeier MA, Cox B, Cohen GN (2013). Clinical outcomes of high-dose-rate brachytherapy and external beam radiotherapy in the management of clinically localized prostate cancer. Brachytherapy.

[8] Forsythe K, Blacksburg S, Stone N, Stock RG (2012). Intensity-Modulated radiotherapy causes fewer side effects than three-dimensional conformal radiotherapy when used in combination with brachytherapy for the treatment of prostate cancer. Int Radiat Oncol Biol Phys.

[9] Spratt DE, Zumsteg ZS, Ghadjar P, Kollmeier MA, Pei X, Cohen G (2014). Comparison of high-dose (86.4 Gy) IMRT vs combined brachytherapy plus IMRT for intermediate-risk prostate cancer. BJU Int.

[10] Hermesse J, Biver S, Jansen N, Lenaerts E, Nickers P (2010). Dosimetric comparison of high-dose-rate brachytherapy and intensity-modulated radiation therapy as a boost to the prostate. Int J Radiat Oncol Biol Phys.

[11] Sobin LH, Compton CC (2010). TNM seventh edition: what’s new, what’s changed: communication from the international Union Against Cancer and the American Joint Committee on Cancer. Cancer.

[12] Yoshida K, Yamazaki H, Takenaka T, Kotsuma T, Yoshida M, Masui K (2014). High-dose-rate interstitial brachytherapy in combination with androgen deprivation therapy for prostate cancer. Strahlenther Onkol.

[13] Deutsch I, Zelefsky MJ, Zhang Z, Mo Q, Zaider M, Cohen G (2010). Comparison of PSA relapse-free survival in patients treated with ultra-high-dose IMRT versus combination HDR brachytherapy and IMRT. Brachytherapy.

[14] Martinez AA, Gonzalez J, Ye H, Ghilezan M, Shetty S, Kernen K (2011). Dose escalation improves cancer-related events at 10 years for intermediate- and high-risk prostate cancer patients treated with hypofractionated high-dose-rate boost and external beam radiotherapy. Int J Radiat Oncol Biol Phys.

[15] Nag S, Bice W, DeWyngaert K, Prestidge B, Stock R, Yu Y (2000). The American brachytherapy recommendations for permanent prostate brachytherapy postimplant dosimetric analysis. Int J Radiat Oncol Biol Phys.

[16] Pinkawa M, Gagel B, Asadpour B, Piroth MD, Klotz J, Borchers H (2008). Seed displacements after permanent brachytherapy for prostate cancer in dependence on the prostate level. Strahlenther Onkol.

[17] Salembier C, Lavagnini P, Nickers P, Mangili P, Rijnders A, Polo A (2007). Tumor and target volumes in permanent prostate brachytherapy: A supplement to the ESTRO/EAU/EORTC recommendations on prostate brachytherapy. Radiother Oncol.

[18] Daskalov GM, Löffler E, Williamson JF (1998). Monte Carlo-aided dosimetry of a new high dose-rate brachytherapy source. Med Phys.

[19] Granero D, Vijande J, Ballester F, Rivard MJ (2011). Dosimetry revisited for the HDR ^192^Ir brachytherapy source model mHDR-v2. Med Phys.

[20] Dale RG (1985). The application of the linear-quadratic dose-effect equation to fractionated and protracted radiotherapy. Br J Radiol.

[21] Botta F, Cremonesi M, Ferrari ME, Amato E, Guerriero F, Vavassori A (2013). Investigation of 90Y-avidin for prostate cancer brachytherapy: a dosimetric model for a phase I-II clinical study. Eur J Nucl Med Mol Imaging.

[22] Ghadjar P, Zelefsky MJ, Spratt DE, Munck af Rosenschöld P, Oh JH, Hunt M (2014). Impact of dose to the bladder trigone on long-term urinary function after high-dose intensity modulated radiation therapy for localized prostate cancer. Int J Radiat Oncol Biol Phys.

[23] Gittelman MC, Marks LS, Hill LA, Volinn W, Hoel G (2010). Effect of silodosin on specific urinary symptoms associated with benign prostatic hyper plasia: analysis of international prostate symptom scores in 2 phase III clinical studies. Open Access J Urol.

[24] Abramowitz MC, Li T, Buyyounouski MK, Ross E, Uzzo RG, Pollack A (2008). The Phoenix definition of biochemical failure predicts for overall survival in patients with prostate cancer. Cancer.

[25] Spradling K, Khoyilar C, Abedj G, Okhunov Z, Wikenheiser J, Yoon R (2015). Redefining the Autonomic Nerve Distribution of the Bladder Using 3-Dimensional Image Reconstruction. J Urol.

[26] Díez P, Mullassery V, Dankulchai P, Ostler P, Hughes R, Alonzi R (2014). Dosimetric analysis of urethral strictures following HDR (192)Ir brachytherapy as monotherapy for intermediate- and high-risk prostate cancer. Radiother Oncol.

